# In Spare Moments

**Published:** 1889-02-09

**Authors:** 


### IN SPARE MOMENTS.

Madame Lisé Monchablon writes: The young friends of my staff unite with me in thanking you for your most kind letter and article in The Hospital of the 19th inst. We are conscious that the outcome of these "spare moments" was very trifling, and fell far short of what we had wished to accomplish. During the present year we hope to be more successful, and if your remarks serve to stimulate others, blessed with more leisure than ourselves, to help in so excellent a cause, it will be a source of gratification to us all. The books were far from what we had intended, as we wished to make them not only untearable and interesting, but also ornamented with scraps and pictures on the blank side of the cloth, but this last idea had to be abandoned, owing to pressure of work which involved late hours, as well as early ones; hence their rather unfinished appearance. We also thank you for so kindly sending these trifles to Maida Vale, and shall feel a great interest in this little institution, of which we have not hitherto heard. Please accept our best wishes for the present year, and that your great hospital work may meet with ever-increasing success.

